# An elementary review on basic principles and developments of qubits for quantum computing

**DOI:** 10.1186/s40580-024-00418-5

**Published:** 2024-03-18

**Authors:** Eunmi Chae, Joonhee Choi, Junki Kim

**Affiliations:** 1 Department of Physics, Korea University, Seoul , 02841 Republic of Korea; 2 Department of Electrical Engineering, Stanford University, Stanford, CA 94305 USA; 3 SKKU Advanced Institute of Nanotechnology (SAINT) & Department of Nano Science and Technology, Sungkyunkwan University, Suwon, 16419 Republic of Korea; 4 Department of Nano Engineering, Sungkyunkwan University, Suwon, 16419 Republic of Korea

**Keywords:** Qubits, Quantum operations, Quantum computers

## Abstract

An elementary review on principles of qubits and their prospects for quantum computing is provided. Due to its rapid development, quantum computing has attracted considerable attention as a core technology for the next generation and has demonstrated its potential in simulations of exotic materials, molecular structures, and theoretical computer science. To achieve fully error-corrected quantum computers, building a logical qubit from multiple physical qubits is crucial. The number of physical qubits needed depends on their error rates, making error reduction in physical qubits vital. Numerous efforts to reduce errors are ongoing in both existing and emerging quantum systems. Here, the principle and development of qubits, as well as the current status of the field, are reviewed to provide information to researchers from various fields and give insights into this promising technology.

## Introduction

Quantum computing is emerging as a ground breaking technology that has attracted widespread attention as the next-generation frontier. The growing complexity of modern technology challenges, particularly in fields such as chemistry, materials science, and finance, has surpassed the capabilities of conventional classical computers. Quantum computing is promising for addressing these challenges, which were previously considered nearly impossible to solve [1–13]. Its significance has become even more pronounced because quantum computers have the potential to revolutionize cryptography [14]. Consequently, numerous countries and organizations are actively exploring and investing in this transformative technology. As a result, quantum computing has made significant strides over the past decade in demonstrating quantum advantages [15, 16], exploring new material phases [17–19], conducting molecular calculations [20], and studying the quantum dynamics of exotic matters [21, 22].

If the physical realization of quantum computers was impossible, the theory of quantum computing and its algorithms would not have attracted much attention despite their high potential and promises [23]. The recent rapid development of quantum computing hardware has reached a stage where simple quantum algorithms and various proofs of concepts can be successfully implemented, which has further accelerated the expansion of the field. However, the successful implementation of practical quantum algorithms requires significantly more computing resources and demands substantial breakthroughs.

Numerous qubit technologies have been proposed and experimentally studied to date, including superconducting circuits [24–26], trapped ions [27–30], Rydberg atoms [31–35], dipolar molecules [36], semiconductors [37–41], nucleus spins [42–46],photons [47–52], etc. Significant progress has been made in pushing the system size and controllability limits, and state-of-the-art systems demonstrate middle-sized machines exhibiting small quantum algorithms [53, 54], quantum error corrections [55–62], and quantum advantages [15, 16]. Despite successful advancements in quantum computing hardware, recent research has uncovered critical challenges in realizing fault-tolerant, scalable quantum computers [63].

In the race to develop quantum computers over the past three decades, the leading platform has been changing over time from one system to another. The question of which platform will be eventually used to implement a practical large-scale quantum computer remains unanswered. It is possible that an entirely novel type of qubit, which has not yet been proposed or realized, could lead to substantial breakthroughs in quantum computing technology.

Numerous efforts are ongoing to enhance existing quantum computing platforms, including strategies to minimize errors by creating logical qubits from multiple physical qubits or establishing quantum connections between quantum computers [59, 62, 64–66]. The overhead required to construct a logical qubit reduces when the error rates of physical qubits become low. For example, it is expected that a few thousands of physical qubits are required to create one logical qubit when their error rates are 0.1% [63]. Therefore, it is crucial to properly engineer and develop physical qubits for constructing scalable and fully error-corrected quantum computers. Ongoing research is actively exploring new quantum computing platforms, including solid-based options comprising quantum dots and silicon, which are known for their seamless integration with existing technologies [67]. Nanotechnology is pivotal for shaping and preserving the delicate quantum properties essential for progress in this domain [68–70].

Numerous reviews have comprehensively detailed existing quantum computing systems [71–73], including articles about material challenges for quantum computers [74, 75]. This review is intended to facilitate researchers from a broad variety of fields, not only physics but also nanotechnology and material sciences, in developing physical qubit systems. In this regard, we summarize the fundamental quantum properties employed in quantum computing, including superposition and entanglement, and the requirements for qubits and quantum operations. In addition, we explore the current status of this field and its applications.

## A brief review of unique features in quantum mechanics

**Fig. 1:**
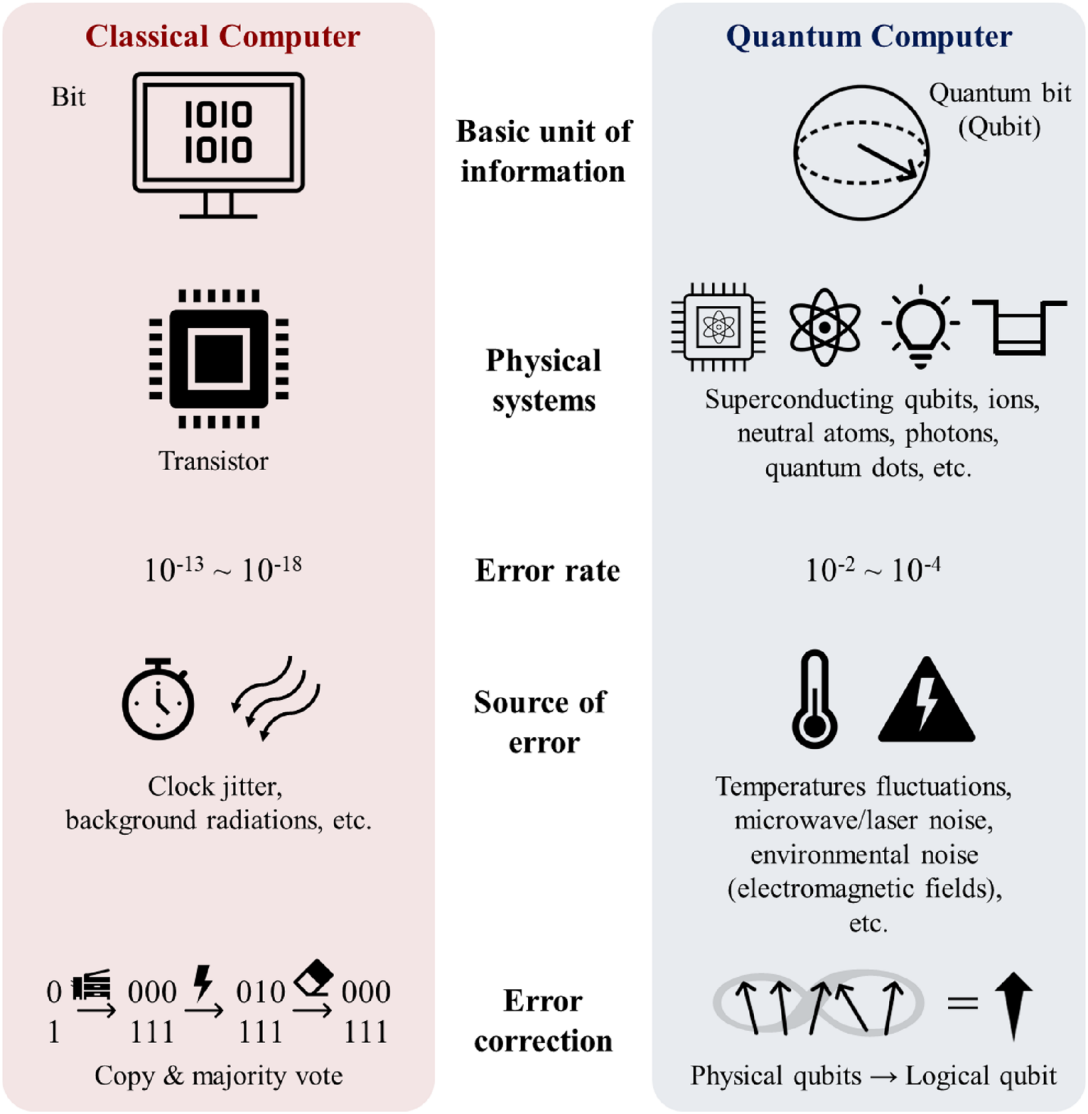
An introductory overview of classical computers and quantum computers. The basic unit of information in classical computers is a digital bit whose value is either 0 or 1. Quantum computers consist of qubits, which have 0 and 1 states, as well as superposition states. The physical system of classical bits is a transistor, while there are many physical systems for qubits. The error rates of classical bits are extremely low, $10^{-13}$–$10^{-18}$, and are caused by clock jitters, background radiation, etc. Temperature fluctuations, noises in microwaves and lasers for qubit manipulation, environmental electromagnetic fields can induce qubit errors at a rate of $10^{-2}$–$10^{-4}$. The errors in classical bits can be corrected by copying the information to multiple bits. Because a quantum state cannot be copied, multiple physical qubits are entangled to form a logical qubit to reduce the error

In this section, we provide a concise overview of the fundamental characteristics distinguishing quantum computers from classical computers (Fig. 1). Quantum computers differ from their classical counterparts by leveraging the principles of ‘superposition’ and ‘entanglement’ of quantum states. Superposition states excessively broaden the possible combinations of qubit states. Currently, typical qubit systems have errors at rates of $10^{-2}$–$10^{-4}$ due to temperature fluctuations, noises in microwaves or lasers used for manipulating qubits, and environmental electromagnetic fields. These errors must be corrected in quantum computations. To minimize errors, it is crucial to entangle multiple physical qubits and form a fault-tolerant ‘logical’ qubit because a quantum state cannot be naively copied, unlike in classical computers. Entanglement is therefore a basic block in the construction of quantum operations. In addition, the stochastic nature of detection in quantum mechanics plays a pivotal role in quantum computing. Each of these aspects is briefly discussed in this section. Many textbooks on quantum mechanics and quantum computations are available for those seeking more detailed explanations [23, 76–78].

### Quantum states and their superposition

In quantum mechanics, the quantum state characterizes the state of a physical system, and contains all information about the system at a given point in time. To construct a quantum computer, qubit states should be defined. For example, the two lowest energy states of a system can constitute a qubit. The two quantum states of qubits are usually denoted $|0\rangle$ and $|1\rangle$ using Dirac notations. Quantum mechanics also permits the system to exist in multiple states simultaneously. This phenomenon is called quantum superposition and can be expressed as a linear combination of two states $a|0\rangle + b|1\rangle$, where *a* and *b* are complex numbers (coefficients) that determine the probability amplitudes of each state. Superposition also results in interference effects, where the probability amplitudes of different states can reinforce or cancel each other out after the state evolution. This interference phenomenon is crucial for quantum algorithms, allowing for the manipulation of probabilities to achieve specific outcomes.

### Entanglement

Entanglement is a multi-particle state that cannot be expressed as the product of two single-particle states. The basis of the quantum states of a system with two qubits, A and B, consists of $|00\rangle$, $|01\rangle$, $|10\rangle$, and $|11\rangle$, where the first number indicates the quantum state of A, and the latter indicates that of B. If measuring the quantum state of A does not affect the state of B, then the two qubits are independent, and we call this the product state of the two. However, if the system is in a state such as $|01\rangle + |10\rangle$, the measurement results for A and B are strongly correlated, and the two particles are entangled.

One of the most exotic aspects of entanglement is its non-local nature. Changes to the state of one entangled particle instantaneously affect the state of the other, regardless of the distance separating them. Some famous examples of entangled states are the Bell states and the Greenberger-Horne-Zeilinger states (GHZ states). The Bell states are four maximally entangled states in a two-particle system with two states: $|0 \rangle$ and $|1 \rangle$. These consist of superpositions of the linear combinations of the two states: $|00 \rangle + |11 \rangle$, $|00 \rangle - |11 \rangle$, $|01 \rangle + |10 \rangle$, and $|01 \rangle - |10 \rangle$. The Bell states are the most basic building blocks in quantum computing processes. A GHZ state, also called the Schrodinger’s cat state, expands the concept of the Bell states to systems with more than two particles. This is a superposition of all 0 qubits and all 1 qubits, such as $|00 \cdots 0 \rangle + |11 \cdots 1 \rangle$.

### Coherence time

Superposition and entanglement are extremely fragile. How long these exotic states are preserved is a measure of the “quantum-ness”, or “coherence”, of a system and determines the quality of a quantum computer. Therefore, measuring the coherence time is important for evaluating a quantum system. Two time scales describe the lifetime of a quantum state: the relaxation time ($T_1$) and dephasing time ($T_2$). $T_1$ is the time scale at which a system relaxes from one quantum state to another, reprensenting a *classical* lifetime. Typically, the $|0\rangle$ state is the ground state of a system, and the $|1\rangle$ state is the first excited state. Owing to the limited lifetime of the excited state, the $|1\rangle$ state relaxes to the ground state $|0\rangle$ after a certain amount of time, as described by $T_1$. Another form of decay affects the phase relationship between the $|0\rangle$ and $|1\rangle$ states, limiting a *quantum* lifetime of a qubit. External factors such as the lifetime of the excited states, temperature, and ambient fields can influence whether a superposition state persists or transforms into the $|0\rangle$ and $|1\rangle$ states. This decay also follows exponential decay and can be characterized by $T_2$, which is the dephasing time. $T_2$ is contingent on the system environment, and its upper limit is determined by $T_1$.

### Detection in quantum mechanics

The nature of measurement in quantum mechanics is very different from that in classical physics. In classical physics, measurements do not affect the measured system. However, quantum measurements destructively affect the quantum state of the system. Let’s assume that our quantum system is in a superposition state of $a|0\rangle + b|1\rangle$. When a measurement is made, the superposition collapses, and the system is observed in one of its possible states, either $|0\rangle$ or $|1\rangle$, with a probability determined by the squared magnitude of the coefficients *a* and *b* in the superposition. This is called “wavefunction collapse”. Therefore, many repeated measurements are necessary to recover all information included in the wavefunction. This probabilistic aspect is a departure from classical determinism.

## Desiderata for qubits

Qubits, the fundamental units of quantum information, are the basic building blocks of quantum computing, and building quantum computers begins with the implementation of physical qubits. Many discussions on the necessary conditions for qubits were conducted in the early era of quantum computing [79–81]. Among these, DiVincenzo proposed a set of five criteria that an ideal qubit should meet to be viable for quantum computing: (i) the ability to initialize the qubit state, (ii) sufficiently long coherence, (iii) the ability to perform universal gate operations, (iv) efficient qubit state readout, and (v) scalability to create large-scale quantum computing architectures [82, 83]. Although the aforementioned criteria are seemingly straightforward, successfully meeting all of these requirements concurrently poses a significant challenge, particularly with regard to scalability.

This section begins by introducing different methodologies for defining qubits in various physical systems. We provide broad approaches to satisfy the requirements of universal quantum computing, focusing particularly on entangling qubits. This section aims to provide guidance for designing and constructing innovative qubits on emerging platforms.

### Defining qubit states

Quantum computers utilize quantum states to store and manipulate information, harnessing properties such as quantum superposition and entanglement. Qubits have two basis states to encode binary quantum information, and an arbitrary amount of quantum information can be stored in a collection of qubits, similar to the classical case.

**Fig. 2:**
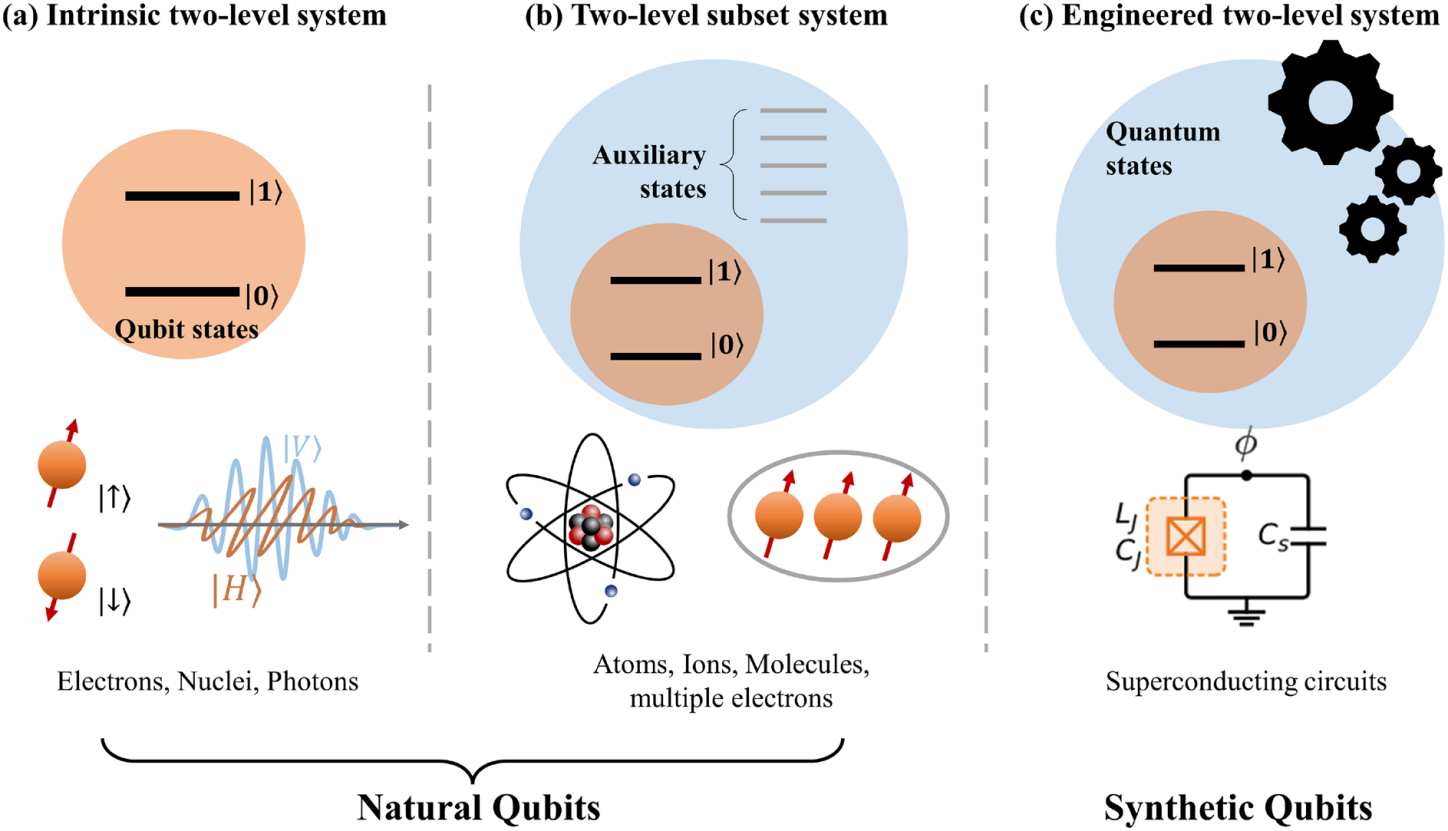
Three different qubit types. Natural qubits employ naturally emerging physical systems such as electrons or atoms. Some systems inherently have two quantum states to be occupied (**a** intrinsic two-level system) while others have a larger state space and the qubit space is defined as a subset (**b** two-level subset system). Synthetic qubits utilize engineered quantum systems and their quantum states can be tuned by system parameters. (**c** engineered two-level system)

Qubit implementations are commonly categorized according to the types of physical platforms used, such as superconducting circuits, neutral atoms, or trapped ions. However, it is equally crucial to consider the choice of quantum states designated as basis states within these physical systems. Physical platforms often contain more than two quantum states and various qubit encodings are possible within a single platform. The properties of the physical qubits are determined not only by the chosen physical system but also by the particular qubit encoding employed within that system. Furthermore, quantum states outside of the defined qubit space can be utilized as resources of quantum operations. Here, we classified qubits into three types: intrinsic two-level systems, two-level subset systems, and engineered two-level systems (Fig. 2).

Because nature is quantum mechanical [84], microscopic physical systems, such as atoms or electrons, naturally exhibit quantum mechanical behaviors. Consequently, these systems can potentially serve as natural qubits. Some systems inherently have two possible quantum states, making them natural candidates for qubits. For example, electrons and some atomic nuclei possess a spin of 1/2, resulting in two possible spin states: up or down. When subjected to a sufficiently large and stable magnetic field, these two spin states become non-degenerate and show long coherence time, which makes them good candidates for qubits. Various physical configurations have been utilized to realize such naturally two-level spin qubits, for instance, electrons floating on liquid helium [85, 86], linear electron Paul trap [87], semiconductor quantum dots [38], nuclear magnetic resonance (NMR) molecules [42, 88] and phosphorus donor atoms in silicon substrate [37, 89]. Polarization of photons is another example of an intrinsic two-level system that has been actively studied as a qubit.

In contrast, atoms or ions are composed of a central nucleus and multiple electrons, forming more complicated systems than a single electron or nucleus. Such composite systems have complex energy levels as an inter-combination of orbital states, spin states of electrons and nuclei, and their couplings [90]; hence, they possess more than two quantum states. A qubit space can be defined as a two-dimensional subset of the Hilbert space, and is classified as a two-level subset system. For example, even when considering the same species of atoms or ions, various types of qubits can be defined such as hyperfine qubits, Zeeman qubits, optical qubits [30], and Rydberg qubits [33], which are named based on transitions between specified qubit states, Color centers such as nitrogen-vacancy centers [91] and multi-electrons confined within a silicon substrate [41] possess more than two possible quantum states, which belong to the category of two-level subset systems.

In two-level subset systems, quantum states may propagate outside the qubit space, resulting in leakage errors during information processing. These errors are particularly problematic because they cannot be corrected using conventional quantum error correction methods [92, 93].

Nevertheless, the presence of auxiliary states can offer certain advantages, as they can serve as intermediate states during information processing. Many qubit control protocols use auxiliary states to implement single- and two-qubit gates [30, 32, 33] and qubit state discrimination and initialization [30, 34, 94]. Despite the aforementioned leakage errors, recent advances in error correction techniques have converted these errors into erasure errors, thereby enhancing the error correction performance [95–98]. Although the presence of redundant quantum states can potentially introduce errors, it can also provide opportunities for engineering advancements if carefully utilized.

Utilizing a high-dimensional Hilbert space with multiple qubits [99, 100] or a single qudit [101, 102], a *d*-dimensional information unit, can be encoded into a single physical system. Certain encoding techniques employ highly entangled states as the basis for the qubits, thereby possessing intrinsic error detection and correction properties [103, 104].

Synthetic qubits, in contrast to natural qubits, utilize the engineered Hilbert space of fabricated quantum systems. For example, the energy eigenvalues, number of bounded states, and other quantum properties of superconducting circuits are highly dependent on the circuit design [25]. A creative design of the Hilbert space may enable realization of high performance qubits in such systems. Conversely, inhomogeneity among numerous qubits can result from imperfections in the fabrication process, whereas natural qubits such as atoms or ions are identical regardless of the qubit counts.

### Manipulation of single-qubit states

The qubit manipulation mechanism depends heavily on qubit states and their platforms because the underlying physical principles are different. To accomplish universal quantum computation with qubits, qubit state initialization, control via single- and two-qubit gates, and state discrimination must be performed [83].

The initialization of qubit states into a fiducial state is a fundamental step in all quantum algorithms and is frequently employed for qubit reuse, particularly for quantum error correction. The initial qubit states generally exist as thermal states with high entropy. A straightforward method for initializing qubits is to cool the qubit systems until they decay to their energy ground state. However, this method requires a sufficiently long wait time to achieve high-fidelity state initialization, making it unsuitable for repeated qubit recycling. Alternatively, quantum states can be transferred to an auxiliary state with a shorter lifetime; for example, the optical pumping process used in trapped ions or neutral atoms [94, 105] or active cooling using microwaves in superconducting qubits [106]. In addition, heralded-state preparation based on state detection can be used to induce rapid high-fidelity state preparation [107, 108].

The control of single-qubit states, i.e., single-qubit gates, can be represented by unitary evolution. A representative example of the single-qubit control method is Rabi oscillation. When these qubits are exposed to a resonant electromagnetic field, the qubit state coherently oscillates between the $|0\rangle$ and $|1\rangle$ states. By tuning the pulse duration, field intensity, and phase, arbitrary single qubit rotations could be realized. For better accuracy and control, more complex configurations such as Raman transitions [109] or stimulated Raman adiabatic passage [110, 111] can be used.

Contrarily, different control methods must be developed when the qubit states are not energy eigenstates or are degenerate. For example, photonic polarization qubits utilize quarter- or half-wave waveplates to realize single-qubit state control [112], and exchange-only qubits of semiconductor quantum dots utilize spin-interaction control via DC voltage pulses on electrodes [113].

The readout of qubit states involves state-dependent interactions with the environments, which commonly rely on spectroscopic methods. For example, in natural atoms or ions, a specific wavelength of light can be applied to drive qubit state-dependent transitions with short lifetimes. By collecting photons from the driven transitions, one can discriminate between the $|0\rangle$ and $|1\rangle$ states using the rate of photon collection [30, 34, 94]. Similar approaches include the state-dependent tunneling of spin qubits [41] and dispersive readout of superconducting qubits [25].

In summary, common strategies are widely applied across numerous qubit platforms, even though qubit manipulation is greatly diversified by physical platforms.

### Creating entanglement between qubits

Quantum entanglement is a key component in quantum computing; however, achieving reliable quantum entanglement can be challenging. Many qubit platforms have successfully demonstrated single-qubit operations but not quantum entanglement. The key challenge is that the qubits should only interact with each other and not with the nearby environment.

**Fig. 3:**
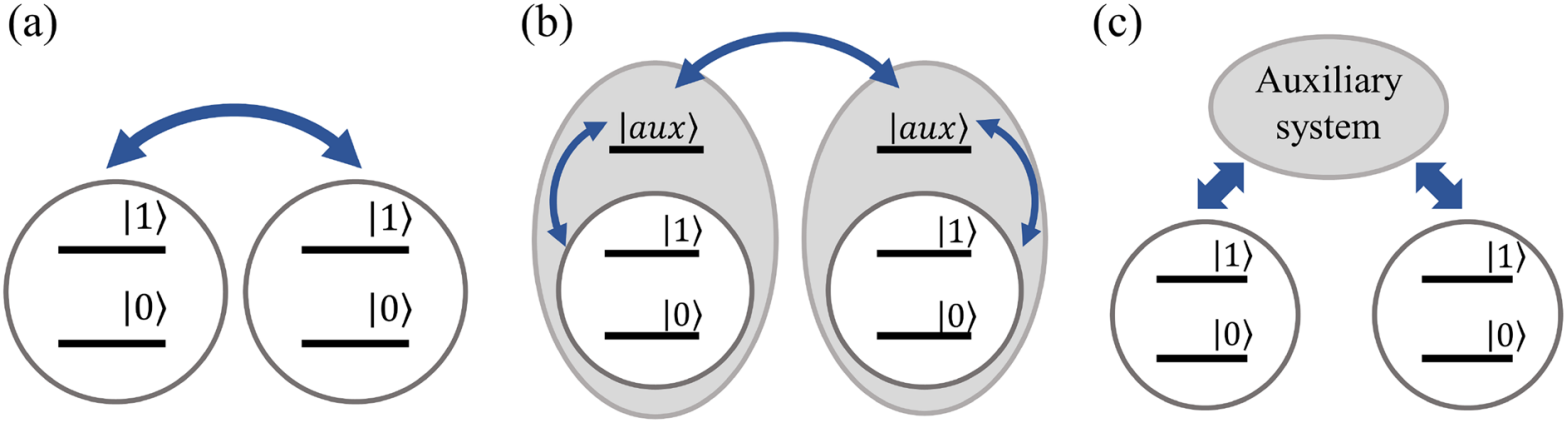
General approaches to establish qubit-qubit entanglements. **a** Some qubit platforms have direct qubit-qubit interactions by nature, which are persistent during the computation process. Controllable interaction can be achieved by introducing **b** auxiliary quantum states or **c** auxiliary quantum systems while maintaining the isolation of qubits. Blue arrows indicate interaction channels between quantum states or systems

Some qubit systems have direct qubit-qubit interaction by their nature, which can be utilized to generate entanglement between qubits (Fig. 3a). Nuclear spin qubits in NMR molecules interact with each other via direct dipolar coupling and indirect through-bond interaction [114]. Using these interactions, various entangling gates and algorithms have been demonstrated for NMR qubits [42–46]. Trapped molecules also exhibit mutual dipolar interaction owing to their large electric dipole moment, but unlike the nuclear spins, their strength can be controlled by varying the distance. By moving molecules trapped in an optical tweezer array, programmable entanglement via spin-exchange interaction among molecule qubits has been demonstrated [115, 116].

Although direct interaction between qubits can be straightforward, it is generally preferable to have controllable interaction rather than persistent interaction. To create entangling interactions while maintaining qubit isolation, (i) auxiliary quantum states (Fig. 3b) or (ii) auxiliary quantum systems (Fig. 3c) can be temporarily introduced to mediate these interactions.

An example of (i) is neutral atomic qubits using Rydberg interaction. Generally, neutral atoms in an optical tweezer array do not interact with each other because of vanishingly small dipole moments. However, when they are excited to highly excited Rydberg states, the enhanced dipole moments induce long-range interactions with nearby atoms, causing the Rydberg blockade phenomenon [31]. By adopting these Rydberg states as auxiliary interaction channels, neutral atoms can store quantum information within low-lying stable quantum states and are temporarily excited to Rydberg states to mediate qubit-qubit entanglement operations [33]. Along with reconfigurable interaction via atomic shuttling, programmable quantum computation has been demonstrated in neutral atom qubit platform [56, 62].

A more broadly applicable approach would be introducing auxiliary quantum systems (ii) that mediate qubit interactions, the so-called “quantum bus”. One example is the collective vibrational (phonon) modes of trapped ion chains. While quantum information is stored in the electronic states of trapped ions, phonon modes as external quantum systems can mediate the entanglement between qubit states. Resonant laser or microwave fields can induce coupling between the electronic and motional states of trapped ions, and by controlling the laser or microwave pulses, qubit-qubit entanglement can be generated in a controllable manner [27, 29, 117, 118]. Similar examples include microwave resonators of superconducting transmon qubits [25] and nitrogen-vacancy centers mediating many carbon-13 nuclei [119]. When auxiliary states or systems are introduced temporarily, those intermediate states must be properly disentangled by the end of the operation. Any residual quantum entanglement between qubit space and the auxiliary space can lead to the decoherence of qubit states and operational errors in quantum computations.

By utilizing and tuning the interactions between qubits, two-qubit unitary evolution or two-qubit entangling gates can be realized. Arbitrary quantum operations or algorithms on qubits can be decomposed into two-qubit entangling gates and single-qubit rotations, which form a universal quantum gate set. Although the controlled-NOT operation is broadly used as a universal gate element, almost any two-qubit entangling gate with single qubit rotations can form a universal gate set [114, 120, 121]. Once qubits are equipped with universal gates, state initialization, and measurement, they can be operated as a universal quantum computer.

## Towards development of a quantum computer

Quantum computers possess disruptive potential across various fields, leveraging quantum parallelism to achieve substantial advantages in both speed and efficiency compared to classical counterparts [1, 2, 9, 15, 16, 122, 123]. Notable examples of the power of quantum parallelism include Shor’s algorithm [124] and Grover’s search algorithm [125]. Specifically, quantum computers’ capabilities in optimization [13], cryptography [14], and scientific research [20, 21] promise transformative breakthroughs in areas such as security [126], finance [10–12], drug discovery [3–6], and material science [7, 8].

While advancements in quantum devices have been remarkable over the past decade, realizing a fully programmable quantum computer capable of running any quantum algorithm, typically referred to as universal quantum computation, is an experimentally challenging task. Despite their potential, the current quantum computing devices are imperfect and susceptible to environmental noise. However, significant strides have been made over the past decade, achieving remarkable single- and two-qubit gate fidelities, surpassing 0.9999 and 0.999, respectively [97, 98, 127].

These advancements naturally raise several timely questions: (1) Can large-scale, albeit ‘noisy’, quantum devices outperform classical ones in specific applications? [15, 16]; (2) How can we verify ‘quantum advantage’? [128, 129]; (3) Can we envision achieving a fault-tolerant quantum computer with error-correcting protocols in the near future? [59, 61, 62]. In the following, we provide a brief summary of these points.

### Analog and digital quantum device

As mentioned earlier, universal quantum computing requires the implementation of arbitrary single-qubit rotation and two-qubit entangling gates, as well as an independent local readout of all qubits. A combination of these elements serves as the building block for preparing and benchmarking arbitrary target states. However, owing to experimental limitations, not all quantum devices fulfill these requirements.

**Fig. 4:**
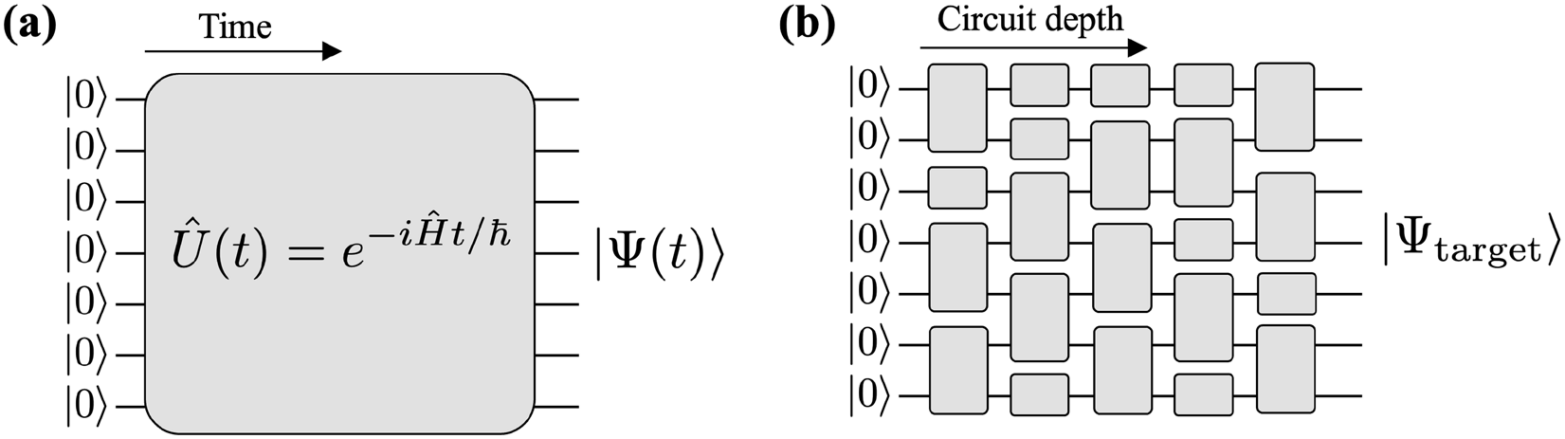
**a** Analog Hamiltonian evolution and **b** digital quantum circuit. In **a**, quench evolution is performed as a function of time under a time-independent Hamiltonian $\hat{H}$, implementing a time evolution operator $\hat{U}(t)$ and thereby transforming the initial all-zero qubit state, $|\Psi (0)\rangle = |00\cdots 00\rangle$, into the target state, $|\Psi (t)\rangle = \hat{U}(t)|\Psi (0)\rangle$, at a later time *t*. In **b**, single- and two-qubit gates are employed to construct a quantum circuit. By applying judiciously chosen quantum gates, the target quantum state, $|\Psi _\text {target}\rangle$, is prepared via digitized state evolution

Despite the experimental constraints and limitations, it is valuable to study the behavior of large quantum systems by evolving an initial state over time under their natural Hamiltonians (Fig. 4a). If these Hamiltonians include strong interactions between qubits, the simple time evolution from a trivial unentangled state, such as with all qubits initialized in the ground state, leads to a generation of entangled states as a result of qubit-qubit interactions. This type of evolution is often referred to as quench dynamics and is employed for *analog quantum simulations* [130]. When the size of a quantum system becomes large or if the amount of entanglement is too extensive at late times, simulating quench dynamics using classical computers can be extremely challenging. For example, the current world-leading record for simulating large-scale quantum dynamics in generic Hamiltonian cases is limited to 38 qubits [131]. Hence, the use of an experimental quantum device for analog quantum simulations provides a way to understand large-scale quantum systems that are difficult to investigate using classical computers.

Specifically, if the Hamiltonian parameters of a given quantum device can be varied to emulate the behavior of another quantum system, for example, by adjusting the external electromagnetic fields or applying a sequence of control pulses, many-body quantum dynamics can be systematically studied in a controlled manner [130]. These direct experimental approaches are particularly valuable for understanding complex quantum phenomena in condensed matter physics, quantum chemistry, and other fields, where the computational demands of simulating quantum behavior on a classical computer become impractical.

In contrast, fully programmable quantum computers have the potential to enable practical applications beyond the reach of their classical counterparts [132]. Unlike analog quantum simulators that rely on continuous-time evolution, a fully programmable quantum computer can employ digital quantum logic (Fig. 4b). Specifically, this involves the utilization of the quantum analogs of logic gates, which serve as building blocks for designing quantum circuits for quantum computations. The diverse applications of universal quantum computers have extended from cryptography to materials science and drug discovery [133].

### Near-term intermediate-scale quantum devices

The best quantum computers reported thus far provide a seemingly high two-qubit gate fidelity, exceeding 0.999. However, the ‘global’ quantum fidelity, defined as the probability of making no errors at the end of a sequence on a system level, decreases exponentially with the number of gates used in the sequence. Such imperfect quantum computers without any error correction have recently been termed Noisy Intermediate-Scale Quantum (NISQ) devices [134]. For instance, Google and a research group at the USTC in China reported global fidelities of approximately 0.002 and 0.0007 for their 53-qubit and 56-qubit quantum computers based on superconducting information processors in 2019 [15] and 2021 [122], respectively. Although their global fidelity values may seem quite low at first glance, they claimed that these global fidelities were sufficiently high to solve certain computationally hard problems much faster than classical computers, marking the first demonstration of quantum advantage. However, these computationally expensive problems intentionally employ randomized quantum circuits consisting of randomly chosen single- and two-qubit gates to create large but random states. These highly entangled, yet random, quantum states were introduced to showcase an example in which quantum computers could outperform classical computers.

In addition to these quantum advantage tests based on random states, there is a growing interest in exploring whether a NISQ device can be employed for more practical problems. While this remains an open question, IBM has recently published a paper on solving the Schrödinger equation for the celebrated quantum Ising model using a 127-qubit quantum computer [123]. In the paper, they demonstrate that physically relevant observables from a large-scale quantum simulation can be accurately measured in situations where brute-force classical computation of the Schrödinger equation becomes intractable due to exponentially long runtimes and large memory sizes. They employed a technique called noise mitigation [135] to predict the ground truth values of the observables of interest. The application of noise mitigation techniques to noisy quantum computers may provide insights into how to better utilize NISQ devices for practical applications, especially in quantum simulations.

### Quantum error correction for fault-tolerant computing

The physical qubits discussed above are inherently susceptible to errors and noise, making error correction crucial for preserving the integrity of quantum information. The goal of fault-tolerant quantum computing is to ensure stable quantum operations, even in the presence of random errors and control imperfections. In summary, this involves the identification of errors in both space and time through syndrome measurements using additional ancillary qubits for real-time error correction [136]. This sophisticated approach to error correction in quantum hardware is inevitable because the existing classical error-correcting protocols are not directly applicable. This is due to the no-cloning theorem [23], which states that it is impossible to create an identical copy of an arbitrary unknown quantum state.

Full details regarding quantum error correction are beyond the scope of this elementary review (see Ref. [137] for more extensive reviews). The main idea was to define multiple delocalized entangled qubits. Specifically, the threshold theorem [23] establishes that if the error rate per physical gate operation is below a certain threshold, the delocalized state can be robustly stabilized through real-time monitoring and error correction using quantum stabilizers [138]. Hence, with the help of quantum stabilizers as spectators of random error events, the delocalized state defined over many noisy qubits can be protected to encode quantum information in an error-resilient form, which is called a ‘logical’ qubit.

Over the last few decades, extensive theoretical and experimental research has been conducted to develop various forms of logical qubits from different physical qubits, which are also referred to as quantum error correction codes. These include Shor’s code [139], Steane code [62], surface code [59, 140], and cat code  [141]. Recently, the experimental realization of logical qubits has been demonstrated in leading quantum platforms, such as superconducting qubits [59], trapped ions [140], and neutral atom arrays [62]. In particular, neutral atom arrays exhibit entanglement operations with up to 48 logical qubits out of the 280 physical qubits, demonstrating a quantum error correction code [62]. This achievement was realized by employing a real-time array reconfiguration based on atom transport, along with zoned architectures for entanglement, readout, and information storage. These results show great promise for realizing medium-scale logical quantum information processors with $\sim$ $10^{2-3}$ logical qubits from $\sim$ $10^4$ physical qubits in the near future [62].

## Conclusion

The basic principles and requirements of qubits and their prospects for quantum computing were reviewed. After demonstrating the precise control of qubits, quantum computers with hundreds of noisy qubits have become readily available in various systems and are proving its value in various fields, including quantum simulations and computations. However, owing to the limitations of current quantum computing systems, developing a fully fault-tolerant quantum computer requires considerable efforts in various areas, particularly in the constituent hardware of qubits. Attempts to identify new qubits will uncover new insights and rejuvenate research and development efforts in the broader field of quantum computing.

## Data Availability

Not applicable.
